# Diagnostic Immunization with Bacteriophage ΦX 174 in Patients with Common Variable Immunodeficiency/Hypogammaglobulinemia

**DOI:** 10.3389/fimmu.2014.00410

**Published:** 2014-08-29

**Authors:** Lauren L. Smith, Rebecca Buckley, Patricia Lugar

**Affiliations:** ^1^Division of Pediatric Allergy and Immunology, Department of Pediatrics, Duke University Medical Center, Durham, NC, USA; ^2^Department of Immunology, Duke University School of Medicine, Durham, NC, USA; ^3^Division of Pulmonary, Allergy, and Critical Care Medicine, Department of Medicine, Duke University Medical Center, Durham, NC, USA

**Keywords:** common variable immunodeficiency, bacteriophage ΦX 174, primary immunodeficiency, hypogammaglobulinemia

## Abstract

**Purpose:** Use of the T cell-dependent neoantigen bacteriophage ΦX 174 has been described since the 1960s as a method to assess specific antibody response in patients with primary immunodeficiencies. We reviewed a cohort of patients at Duke University Medical Center who received immunization with bacteriophage and report the clinical utility and safety of the immunization, as well as patient characteristics.

**Methods:** A retrospective chart review was performed of all Duke Immunology Clinic patients (pediatric and adult) who received immunizations with bacteriophage, from 1976 to 2012. Subjects were selected for inclusion if their diagnosis at the time of bacteriophage was either presumed or confirmed common variable immunodeficiency (CVID), hypogammaglobulinemia, transient hypogammaglobulinemia, or antibody deficiency unspecified. Follow up post-immunization was also recorded.

**Results:** One hundred twenty-six patients were identified, 36 adults and 90 pediatric patients. Diagnoses prior to bacteriophage were CVID (*n* = 100), hypogammaglobulinemia (*n* = 23), and antibody deficiency (*n* = 3). Post-immunization diagnoses were CVID (*n* = 65), hypogammaglobulinemia (*n* = 19), unknown (*n* = 23), no primary immune deficiency (*n* = 10), and other primary immunodeficiency (*n* = 9). Seventy-five patients had abnormal bacteriophage results, 37 were normal, and 14 were borderline. There were 257 recorded administrations of the immunization. Information was available on adverse reactions for 171 administrations. Fourteen immunizations were associated with minor adverse events. Nineteen patients stopped their immunoglobulin replacement therapy based on reported normal responses to immunization.

**Conclusion:** Bacteriophage ΦX 174 immunization is a safe, well-tolerated, and clinically useful method to assess antibody response in patients with suspected antibody-mediated immunodeficiencies, particularly those who are on immunoglobulin replacement therapy at the time of immunization.

## Introduction

Bacteriophage ΦX 174 is a T cell dependent neoantigen first described by Uhr et al., where it was used to assess antibody production in guinea pigs (1). This “potent antigen” only infects its host strain of *E. Coli* C and not mammalian cells. When injected intravenously, two methods of assessment are used to determine its effect: clearance of phage from the blood and *in vitro* measurement of phage neutralization by IgM antibodies after the primary immunization and by IgG antibodies after the second immunization (2). In 1966, Ching et al. described the use of the immunization to assess eight children with hypogammaglobulinemia and compare them to healthy controls (2). They were able to assess clearance of phage from the bloodstream, as well as IgM and IgG antibody responses (2). Antibody production is measured as a logarithmic neutralization factor, or *K* value (Kv), which represents the rate of inactivation of phage (1, 3). A majority of the eight patients they evaluated had responses of reduced magnitude, as well as defects in isotype switching (2).

In 1971, Ochs et al. evaluated 26 patients with various types of primary immunodeficiency, including eight with antibody deficiencies, by injecting them with bacteriophage ΦX 174 (3). Most patients received at least a primary and secondary immunization; many also received tertiary and quaternary immunizations to study their response (3). They were compared to normal controls. Of the patients with unspecified antibody deficiency, the majority had depressed responses to phage, little, or no demonstrable isotype switch from IgM to IgG after secondary or tertiary immunizations, and required immunoglobulin replacement therapy (3).

In 1975, Wedgwood et al. reported their experience with bacteriophage ΦX 174 immunization for immune assessment of specific antibody production as “the single most useful antigen for the systemic study of antibody responses in man.” (4) They further classified the “normal” response, as well as classifying the abnormal response into Types 0 through 5, based upon the antibody amount, immunoglobulin class produced, and memory amplification in the primary and secondary responses. The same group was further able to characterize phage responses by developing an ELISA technique, comparable to the neutralization assay, which allowed them to directly measure immunoglobulin isotypes and specific antibody subclasses (5). They confirmed the normal sequence of immunoglobulin class antibody responses to immunization with phage, as well as the characteristic memory response, amplification, and isotype switching that occur after secondary and tertiary immunization (5). More recently, the response to immunization with bacteriophage ΦX 174 in 10 patients diagnosed with adenosine deaminase deficiency (ADA) before and after various treatments was evaluated for specific antibody responses after treatment. The authors determined that patients treated with bone marrow transplantation or PEG–ADA showed improvement in their bacteriophage specific antibody response, as opposed to patients treated with red blood cell transfusions, who continued to exhibit severely depressed responses (6). In addition, Buckley et al. have used bacteriophage immunization response as a means to evaluate B cell function in post-transplantation for severe combined immunodeficiency (SCID) patients, thus allowing for a more definitive post-transplant treatment plan (7).

Common variable immunodeficiency is a clinical syndrome that likely includes many different genetic defects and has a broad spectrum of clinical and laboratory manifestations. It is characterized by the presence of low or absent serum immunoglobulin G and IgA and/or IgM despite the presence of circulating B cells. Patients with common variable immunodeficiency (CVID) may have a constellation of clinical findings including recurrent infections, autoimmunity, predilection toward certain malignancies, and lymphoproliferation of predominantly the lung and/or gastrointestinal tract (8, 9). Currently, the Pan-American Group for Immunodeficiency (PAGID) and the European Society for Immunodeficiencies (ESID) define probable CVID as “serum IgG and IgA at least two SDs below the mean with the following criteria: (1) onset of immunodeficiency at >2 years of age; (2) absent isohemagglutinins and/or poor response to vaccines; (3) defined causes of hypogammaglobulinemia have been excluded” (10). More recently, revised diagnostic criteria have been proposed with a greater emphasis on clinical presentation, as well as presenting criteria for initiating immunoglobulin replacement (11).

Bacteriophage ΦX 174 is still considered experimental and requires an investigational new drug (IND) approval and an institutional review board approved protocol for its administration. However, it is the only neoantigen that has been well studied to assess antibody responses. The fact that it is a neoantigen allows for the appropriate evaluation of patients with a suspected immune deficiency, in particular CVID, who are already on immunoglobulin replacement therapy. This is due to the lack of natural protective antibody to bacteriophage ΦX 174 in humans, therefore not present in commercial immunoglobulin replacement products. Furthermore, the geography of the pooled donors does not weigh into consideration for bacteriophage immunization (12).

We performed a retrospective chart review of all Duke Immunology Clinic patients (pediatric and adult) who received immunization with bacteriophage, from 1976 to 2012. Subjects were selected for inclusion if their diagnosis at the time of bacteriophage was either presumed or confirmed CVID, hypogammaglobulinemia, transient hypogammaglobulinemia, or antibody deficiency unspecified. Our primary goal was to report clinical utility and safety of the immunization, as well as the patient characteristics of our cohort.

## Materials and Methods

All patients received bacteriophage ΦX 174 immunization (FDA number BB-IND 5620) after receiving informed consent for investigational use in accordance with Duke University Medical Center institutional review board approval and protections for human subject research considerations. Patients were immunized with 0.022 mL/kg of bacteriophage ΦX 174 given intravenously. Peripheral blood was collected immediately prior to the first immunization, 15 min after the first immunization and thereafter at 7, 14 days, and 4 weeks later to assess the primary antibody response. A second immunization was given 6–10 weeks after the primary immunization per protocol. A peripheral blood sample was collected prior to the second immunization. The dose of the second immunization was given IV at 0.022 mL/kg just as the primary, with peripheral blood samples collected at 7 and 14 days after the second immunization and then again at 4 weeks after. Two immunizations formulated the standard protocol for antibody assessment, although some patients did receive tertiary immunization 6 months to years later as part of ongoing evaluations.

All peripheral blood samples were sent to the Immunology Diagnostic Laboratory at Seattle Children’s for determination of phage clearance, antibody neutralization, and isotype switch response prior to and after immunization. Bacteriophage samples were processed as previously described by Wedgwood et al. (4). When given intravenously, bacteriophage stays in the intravascular space until clearance occurs (4). The agar overlay method is used on the various samples to determine the number of plaque-forming units (PFU) of phage per milliliter of serum and therefore assess bacteriophage clearance (4). To assess antibody production, either a neutralization assay or ELISA is used. The neutralization assay incubates a standard amount of bacteriophage with diluted serum and uses the agar overlay method at specific intervals to determine the amount of residual bacteriophage (4). The Kv (rate of inactivation) is calculated using a standard formula (4). Isotype switch response is determined using 2-mercaptoethanol susceptibility; gel filtration demonstrates a macroglobulin peak (IgM) and another peak representative of IgG (4).

We performed a retrospective chart review on all the patients who have been immunized with bacteriophage at our institution, selecting the patients whose reason for having bacteriophage performed was listed as CVID, hypogammaglobulinemia, transient hypogammaglobulinemia, or antibody deficiency. Dates of immunization ranged from 1976 to 2012.

## Results

### Patient demographics

One hundred twenty-six patients were identified. At the time of immunization, 90 patients were pediatric and 36 were adults. Prior to immunization with bacteriophage, 100 patients had a diagnosis of CVID, 23 had a diagnosis of hypogammaglobulinemia (transient or other), and 3 had a diagnosis of antibody deficiency (Table 1).

**Table 1:** **Demographics**.

Diagnosis	Pre-test diagnosis (*n* = 126)		Post-test diagnosis (*n* = 126)	
	Total	Pediatric (%)	Total	Pediatric (%)
CVID^a^	100	65∕100 (65)	65	44∕65 (68)
Hypogammaglobulinemia (transient or other)	23	22∕23 (96)	19	15∕19 (79)
Antibody deficiency NOS	3	3∕3 (100)	0	–
No PID	−		10	3∕10 (30)
Other^b^	−		9	7∕9 (78)
Unknown	−		23	20∕23 (91)

*^a^Diagnosis of CVID as listed in the medical record – 44 patients met ESID criteria for CVID at the pre-test diagnosis, 38 did not, and 44 had incomplete data to assess*.

*^b^Other, hyper IgM syndrome, IgA deficiency, PNP deficiency, CID, and unspecified PID*.

**Figure 1:**
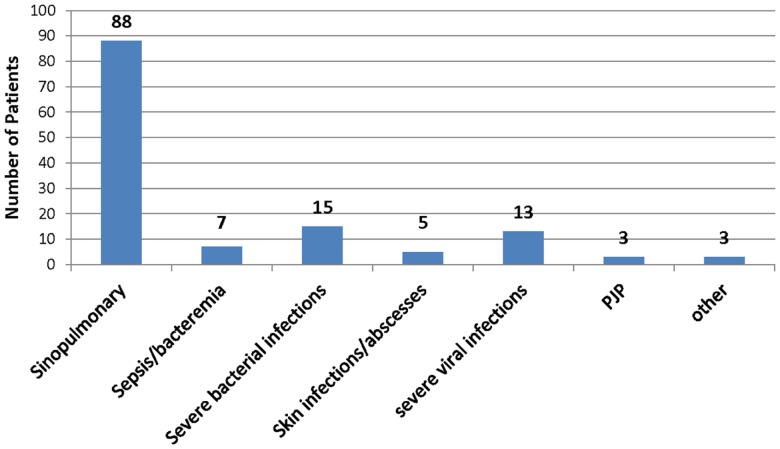
**Infection data historical infections in study patients**. One hundred nine patients’ records were complete to evaluate relevant infection history. The types of infections are shown among the patients evaluated with bacteriophage immunization. See text for greater detail.

Complete clinical history for interpretation was available for 109 of the 126 patients. All subject records noted recurrent infections of varying severity. Eighty-eight subjects had recurrent sinopulmonary infections. Seven had sepsis and/or bacteremia. Fifteen had severe bacterial infections, which consisted of pericarditis, meningitis, severe pneumonia, septic arthritis, osteomyelitis, psoas abscess, and complicated otitis media. Five subjects had skin infections or abscesses noted in their clinical history. Thirteen had severe viral infections, consisting of recurrent HSV, EBV, severe VZV, RSV requiring intubation, death from disseminated enterovirus, and severe warts. Three patients had PJP pneumonia, and three patients had pathogenic infections due to other less common infectious organisms, including candida and strongyloides (Figure 1).

### Laboratory features

Immunoglobulin values were recorded from the time the patient received bacteriophage, or, if available, from the earliest patient encounter available. For some patients, the only immunoglobulin values available were obtained after immunoglobulin replacement therapy had already been initiated. Forty-five patients had an IgG <300 mg/dL; one of these was on replacement therapy. Forty-five patients had IgG levels from 300 to 499; three of those patients were on replacement therapy. Fourteen patients had IgG levels from 500 to 699; four of those were on replacement therapy. Twenty patients had IgG >700; 12 of those were on replacement therapy. Two patients did not have immunoglobulin levels available. Other than the 20 patients known to be on replacement therapy, all values were obtained prior to initiation of IV or subcutaneous Ig. Ninety patients had baseline IgG at or under 499 mg/dL.

In the group of patients with IgG <300, 42/45 had either IgA or IgM below two SDs for age. In the group with IgG from 300 to 499, 38/45 had low IgA or IgM. In the group from 500 to 699, 9/14 had concomitant low IgA or IgM. In the group with IgG >700, 11/20 had low IgA or IgM levels (Table 2).

**Table 2:** **Serum immunoglobulin levels prior to immunization**.

Parameter	Patients (*n* = 126)	Low IgA or IgM	On Ig replacement^a^	Abnormal phage (*n* = 75)	Borderline phage
IgG <300	45	42	1/45	31	7
IgG 300–499	45	38	3/45	24	5
IgG 500–699	14	9	4/14	9	0
IgG >700	20	11	12/20	10	2
Ig not available	2	−	–	1	0

*^a^Number of patients on Ig replacement when immunoglobulins were drawn*.

Eighty-eight patients had *in vitro* studies of lymphocyte proliferation in response to both mitogen and antigen stimulation performed. Of those, 10/88 had abnormalities in both mitogens and antigens, 1 had an abnormal response to mitogens only, 19 had abnormal responses to antigens, and 58 were normal. Twelve patients had proliferation performed only in response to mitogens, and of those, 4/12 were abnormal (Table 3).

**Table 3:** **Lymphocyte proliferation**.

Parameter	Patients	Abnormal phage	Borderline phage
Lymphocyte proliferation with both mitogens and antigens	88	−	–
Both abnormal	10	9	1
Mitogens abnormal	1	1	0
Antigens abnormal	19	8	4
Both normal	58	35	6
Only mitogens performed	12	6	1
Abnormal mitogens	4	3	0
Only antigens performed	0	−	–

*Lymphocyte proliferation. Shows the number of patients who had lymphocyte proliferation studies done and bacteriophage ΦX 174 results. Mitogens tested were phytohemagglutinin (PHA), concanavalin A (ConA), and pokeweed mitogen (PWM). Antigens tested were tetanus and candida*.

### Diagnoses and bacteriophage results

**Figure 2:**
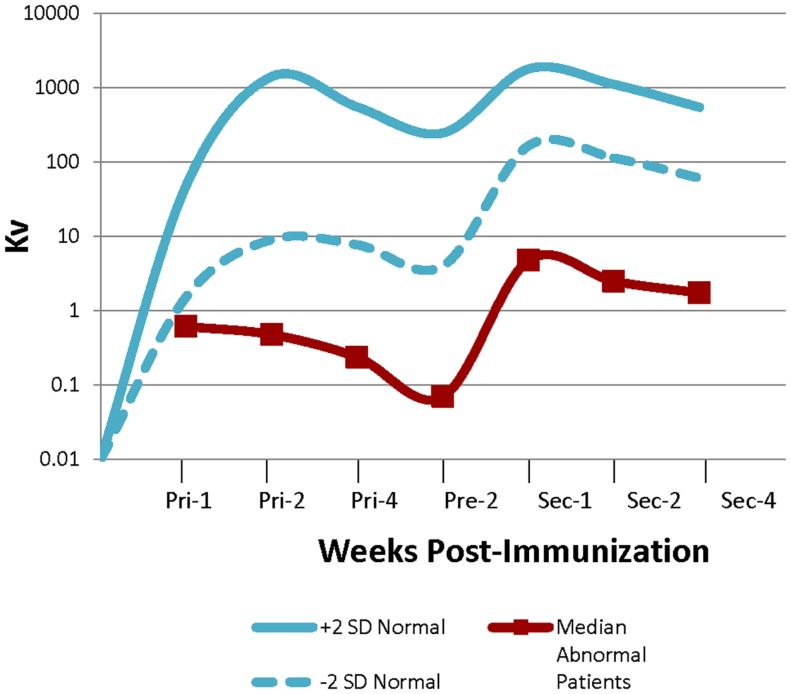
**Abnormal bacteriophage ΦX 174 results bacteriophage immunization results**. Graph showing the range of normal bacteriophage (±2 SDs) and the median values for the abnormal patients. Values were obtained 1, 2, and 4 weeks after the primary immunization, prior to administering the secondary, and then 1, 2, and 4 weeks after administration of the secondary immunization (4).

Out of the 126 patients, 37 had normal bacteriophage antibody responses following primary and secondary immunizations, 75 were abnormal, and 14 patients had borderline results. The median Kv (representative of the rate of inactivation of bacteriophage) of the abnormal patients was well below the range for normal controls (Figure 2). No patient had abnormal clearance of bacteriophage ΦX 174. Patients were classified as borderline if their response was described as “near normal” or if their Kv and/or percentage of IgG post-secondary immunization were at the lowest limit of cutoff for a normal result.

Failure to make an appropriate amount of IgG after secondary immunization indicates an inability to isotype switch from IgM to IgG (3). Of the 75 abnormal patients, 38/75 exhibited both low total antibody amounts as well as abnormal ability to isotype switch from IgM to IgG after secondary immunization. Twenty-four of those 38 made zero IgG after the secondary immunization. Thirty-six of the 75 abnormal patients had low total antibody amounts, but they were able to adequately isotype switch from IgM to IgG. One patient out of the 75 had a quantitatively normal amount of antibody but failed to isotype switch (Table 4).

**Table 4:** **Isotype switching in abnormal bacteriophage patients**.

Parameter	Low total antibody and abnormal isotype switching (38/75)	Made 0% IgG (24/38)	Low total antibody but able to switch to IgG (36/75)	Normal total antibody but abnormal isotype switching (1/75)	Number on Ig replacement
**ABNORMAL BACTERIOPHAGE ΦX 174 *N* = 75**					
IgG <300	12	8/12	18	1	0/31
IgG 300–499	12	7/12	12	0	3/24
IgG 500–699	7	3/7	2	0	2/9
IgG >700	6	5/6	4	0	6/10
IgG not available	1	1/1	0	0	0/1

*Isotype switching. Breakdown of the 75 patients with abnormal bacteriophage results describing defect in antibody production, isotype switching, or both*.

After the results of the bacteriophage immunizations were known, 65 patients were diagnosed as having CVID, 19 were diagnosed as having hypogammaglobulinemia (transient or other), 23 were unknown, 10 were determined to have no primary immunodeficiency, and 9 had a diagnosis of “other” (Table 1). “Other” included Hyper IgM syndrome, IgA deficiency, PNP deficiency, combined immunodeficiency, and unspecified primary immune deficiency. The 23 patients who were unknown were simply lost to follow up or had no records available; it is possible that they also carried diagnoses of CVID. Nineteen patients (15%) who had been on immunoglobulin replacement had it discontinued by their treating physician after a normal bacteriophage result was obtained.

We further grouped bacteriophage results in terms of immunologic parameters. Of the 45 patients with IgG <300, 31 (69%) were abnormal and 7 (16%) were borderline. Of the 45 patients with IgG levels from 300 to 499, 24 (57%) were abnormal and 5 (11%) were borderline. Of the 14 patients with IgG 500–699, 9 (64%) were abnormal and 0 were borderline. Finally, of the 20 patients with IgG >700, 10 (50%) were abnormal and 2 (0.1%) were borderline. Of note, 12/20 patients in this group were on immunoglobulin replacement therapy at the time their IgG level was drawn, likely accounting for their normal IgG levels but abnormal response (Table 2).

It is also helpful to look at patient bacteriophage ΦX 174 immune response in relation to recorded patient antibody response to traditional immunizations. Out of the 75 patients with abnormal bacteriophage results, 56 had baseline tetanus and diphtheria antibody titers recorded prior to initiation of immunoglobulin replacement. For tetanus, 45/56 (80%) had abnormal baseline titers. Of those 45, 23 received a booster vaccination, and 19/23 had abnormal titers post-booster vaccination. For diphtheria, 48/56 (86%) had abnormal baseline titers, 23 received booster vaccination, and 19/23 as well remained abnormal after receiving the booster. Only 18 patients with abnormal bacteriophage had data available regarding pneumococcal titers. Of those, 15/18 were abnormal at baseline, 13 received Pneumovax, and 10/13 continued to have low titers after booster vaccination (Table 5).

**Table 5:** **Recorded specific antibody titers in patients with abnormal bacteriophage results (*n* = 75)^a^**.

Parameter (antibody titers)	Number patients with data available	Abnormal baseline	Received booster	Abnormal after booster
Tetanus	56/75	45/56	23	19/23
Diphtheria	56/75	48/56	23	19/23
Pneumococcal	18/75	15/18	13	10/13

*^a^Values obtained prior to initiation of Ig replacement*.

### Safety

Data were available on adverse reactions for a smaller cohort of patients. One hundred twenty-six patients received primary and secondary immunization, and 13 patients also received a tertiary immunization, representing 265 recorded administrations of bacteriophage ΦX 174 immunization. Data were recorded regarding immediate reactions (within 24 h after immunization) for 171 out of 265 administrations of the immunization, while for delayed reactions (those occurring 24–72 h after the immunization) data were available for 53 out of 265 administrations. There were a total of four immediate reactions recorded with administration of bacteriophage: rash (1/171), headache (1/171), and fever (2/171). Therefore, 167/171 (94%) administrations of bacteriophage had no immediate reaction noted.

There were a total of 10 patients reporting delayed adverse events, frequently consisting of multiple discrete symptoms associated with a single immunization. Each discrete symptom was counted toward total adverse events in the delayed period. Thus for 53 administrations of bacteriophage, delayed symptoms included rash (2/53), fever (4/53), itching (2/53), nausea/vomiting (2/53), sweats (1/53), and headache (4/53) times. Therefore, 43/53 (81%) administrations of bacteriophage were tolerated without a delayed reaction after immunization.

Data on adverse reactions are limited by the retrospective nature of this study. Information on reactions was obtained from patient questionnaires that were given out at the time of immunization and then requested to be mailed back. This is likely the primary reason that data were available for only 65% of the administrations of the vaccine. We are unable to say whether the patients who did not return questionnaires experienced adverse events, but in the cohort that we have available the rate seems quite low and the effects mild.

## Discussion

To the best of our knowledge, this is the largest reported cohort of patients with suspected antibody-mediated immunodeficiency who received diagnostic immunization with bacteriophage ΦX 174. This large report additionally shows results of bacteriophage ΦX 174 immunization across both pediatric and adult patients. There were no relevant differences in the results or safety data between pediatric and adult populations. All of the subjects in the study underwent bacteriophage ΦX 174 immunization with a clear indication for an immunologic evaluation based on histories of recurrent or severe infections. Eighty-eight patients (70%) had a documented history of sinopulmonary infections. Sinopulmonary infections are the most frequent type of infection reported in CVID, and often are the presenting symptom and impetus for evaluation (13). Fifteen patients (12%) had severe bacterial infections, and 13 (10%) had severe viral infections. Severe viral infections are not typically associated with CVID. The patient who died from disseminated enteroviral infection was diagnosed with an unspecified primary immunodeficiency. Two patients with severe varicella as well as HSV and EBV, respectively, had confirmed diagnoses of CVID and normal *in vitro* lymphocyte proliferation responses. Another patient with severe varicella was also diagnosed as having CVID, but that patient had abnormal proliferative response to mitogens and antigens. A fourth patient with severe varicella was also diagnosed with CVID and, although he had normal *in vitro* proliferative responses to PHA and ConA, his responses to pokeweed mitogen, tetanus, and candida were low. The patient with warts was diagnosed with CVID and had no T cell proliferation abnormalities. The patient with severe RSV requiring intubation was eventually diagnosed as having transient hypogammaglobulinemia of infancy, which is not unreasonable as RSV is known to potentially cause severe disease in immunocompetent hosts. Only 109 patients had data available on their infectious history, so it is possible that more of our cohort had sinopulmonary infections and other infectious complications.

Although administration of bacteriophage ΦX 174 is labor intensive and requires an IRB approval for investigational use, it is a clinically useful diagnostic tool. Many of the patients in our cohort had a diagnosis of CVID confirmed by abnormal bacteriophage results. Of the 65 patients who had a confirmed post-test diagnoses of CVID, 54 (83%) had abnormal phage results. Six (9%) were borderline. Five patients (8%) were technically listed as having normal results, but 3/5 were atypical with respect to the expected kinetics of the antibody response or proportion of isotype switch. One of those patients had an abnormal 4-week primary response but a normal secondary and was overall considered normal. However, this patient was also noted to have splenomegaly and immune thrombocytopenic purpura, in addition to low immunoglobulins and recurrent infections. Another subject was resulted as normal due to a quantitatively normal overall response, but the primary response was delayed and the proportion of isotype was skewed to a predominant IgM secondary response. A third “normal” patient in the group had normal testing when measured by ELISA, but when the test was performed by neutralization her primary response was mildly low. The final two reported normal results later confirmed diagnoses of CVID that had progressed from IgA deficiency to CVID. Therefore, it is possible that the bacteriophage testing occurred too early in the disease process and thus if performed later or repeated might have yielded abnormal responses. In these examples, immunization with bacteriophage ΦX 174 appears to highlight the utility of confirming whether or not an antibody-mediated immune disorder is present.

Perhaps, the most useful application of bacteriophage ΦX 174 is in assessing patients who are already receiving immunoglobulin replacement therapy. Since bacteriophage is a neoantigen, therapeutic immunoglobulin preparations do not contain natural antibodies to bacteriophage ΦX 174, and therefore immunoglobulin replacement therapy does not need to be discontinued in order to perform the test. At times patients and physicians are forced to choose between continuing a costly treatment that may be unnecessary or stopping it with the risk that it may be essential for keeping the patient healthy, in order to complete an accurate assessment of their immune function. Nineteen patients in our cohort had their immunoglobulin replacement therapy stopped after having normal bacteriophage results. As bacteriophage is a T cell-dependent neoantigen, a normal result does not necessarily mean that the patient has adequate polysaccharide response, and it is possible that IVIG may still be warranted. However, it can be used as a discriminating diagnostic tool and may provide reassurance about the relative safety of stopping Ig replacement therapy.

## Conflict of Interest Statement

The authors declare that the research was conducted in the absence of any commercial or financial relationships that could be construed as a potential conflict of interest.
